# Synthetic Peptides to Target Stringent Response-Controlled Virulence in a *Pseudomonas aeruginosa* Murine Cutaneous Infection Model

**DOI:** 10.3389/fmicb.2017.01867

**Published:** 2017-09-27

**Authors:** Daniel Pletzer, Heidi Wolfmeier, Manjeet Bains, Robert E. W. Hancock

**Affiliations:** Department of Microbiology and Immunology, Centre for Microbial Diseases and Immunity Research, University of British Columbia, Vancouver, BC, Canada

**Keywords:** Stringent response, Anti-biofilm peptides, *Pseudomonas*, mouse abscess infections

## Abstract

Microorganisms continuously monitor their surroundings and adaptively respond to environmental cues. One way to cope with various stress-related situations is through the activation of the stringent stress response pathway. In *Pseudomonas aeruginosa* this pathway is controlled and coordinated by the activity of the RelA and SpoT enzymes that metabolize the small nucleotide secondary messenger molecule (p)ppGpp. Intracellular ppGpp concentrations are crucial in mediating adaptive responses and virulence. Targeting this cellular stress response has recently been the focus of an alternative approach to fight antibiotic resistant bacteria. Here, we examined the role of the stringent response in the virulence of *P. aeruginosa* PAO1 and the Liverpool epidemic strain LESB58. A Δ*relA*/Δ*spoT* double mutant showed decreased cytotoxicity toward human epithelial cells, exhibited reduced hemolytic activity, and caused down-regulation of the expression of the alkaline protease *aprA* gene in stringent response mutants grown on blood agar plates. Promoter fusions of *relA* or *spoT* to a bioluminescence reporter gene revealed that both genes were expressed during the formation of cutaneous abscesses in mice. Intriguingly, virulence was attenuated *in vivo* by the Δ*relA*/Δ*spoT* double mutant, but not the *relA* mutant nor the Δ*relA*/Δ*spoT* complemented with either gene. Treatment of a cutaneous *P. aeruginosa* PAO1 infection with anti-biofilm peptides increased animal welfare, decreased dermonecrotic lesion sizes, and reduced bacterial numbers recovered from abscesses, resembling the phenotype of the Δ*relA*/Δ*spoT* infection. It was previously demonstrated by our lab that ppGpp could be targeted by synthetic peptides; here we demonstrated that *spoT* promoter activity was suppressed during cutaneous abscess formation by treatment with peptides DJK-5 and 1018, and that a peptide-treated *relA* complemented stringent response double mutant strain exhibited reduced peptide susceptibility. Overall these data strongly indicated that synthetic peptides target the *P. aeruginosa* stringent response *in vivo* and thus offer a promising novel therapeutic approach.

## Introduction

Antibiotics are arguably the most successful medical intervention in human history. The lack of effective antibiotics would have devastating effects in medicine such as causing death after major surgeries and even minor injuries. Indeed, resistance to most antibiotics has become a global threat to public health and strategies to overcome this danger are urgently needed. Life-threatening situations with pathogens that are resistant to several classes of antibiotics are on the rise and the widespread distribution of those organisms is of great concern (Ventola, 2015). Finding novel targets to treat bacterial infections has attracted considerable interest, and as such, inhibition of the stringent stress response has been suggested to be a promising alternative approach for disarming pathogens without killing them (Hauryliuk et al., 2015). Moreover, the stringent stress response pathway is a ubiquitous process among bacteria and, importantly, mammalian hosts lack this pathway (Pollard et al., 1980), indicating that the stringent response is an excellent selective drug target in bacteria.

The bacterial stringent response is a crucial survival response to various environmental stress conditions (Potrykus and Cashel, 2008) encountered, for example, during nutrient starvation (Haseltine and Block, 1973), fatty acid or iron limitation (Vinella et al., 2005), heat shock (Vogt et al., 2011), and high density populations such as stationary phase growth (Mansour et al., 2016) and biofilm formation (Strugeon et al., 2016). As a consequence, cells rapidly trigger a cellular reprogramming response (Starosta et al., 2014) that causes bacteria to decelerate energy-consuming processes (such as macromolecular synthesis and growth) and redirect resources toward energy generation, stress coping and expression of biosynthetic genes (Traxler et al., 2008). Activation of the stringent stress response pathway occurs through expression of the genes responsible for the synthesis of the small signaling nucleotide guanosine-pentaphosphate (pppGpp) that is quickly hydrolysed to the active form guanosine-tetraphosphate (ppGpp). In Gram-negative bacteria, two highly conserved homologous proteins, RelA and SpoT, regulate the intracellular concentrations of ppGpp (Pletzer et al., 2016). RelA is a mono-functional synthase that binds to the ribosome and upon entry of an uncharged tRNA molecule that blocks the ribosome, converts GTP and ATP to pppGpp and hence ppGpp. Conversely, SpoT is a bi-functional enzyme that responds to many other types of stresses such as carbon, phosphorus, fatty acid or iron starvation, and triggers the synthesis of ppGpp. Additionally, SpoT possesses another functional domain that allows it to also hydrolyze ppGpp. The second messenger ppGpp functions as a pleiotropic regulator to coordinate the stress response. Rapid accumulation of intracellular ppGpp triggers a switch from cell growth to survival mode through several mechanisms including binding and altering of the specificity of RNA polymerase, interaction with proteins involved in translation, replication and RNA turnover, crosstalk with other second messenger molecules such as c-di-GMP, and by regulation of cellular processes such as quorum sensing (Romling and Balsalobre, 2012; Chua et al., 2015; Hauryliuk et al., 2015; Pletzer and Hancock, 2016).

Although more than 1,000 articles have addressed the stringent response since its discovery 48 years ago (Cashel and Gallant, 1969), only a handful have addressed the bacterial stringent response in the context of vertebrate infection models. Generally speaking, stringent response mutants often exhibit attenuated virulence and persistence as well as enhanced survival rates in a variety of Gram-positive (Kazmierczak et al., 2009; Frank et al., 2014; Mansour et al., 2016; Zhu et al., 2016), mycobacterial (Klinkenberg et al., 2010; Weiss and Stallings, 2013), and Gram-negative bacterial infections (Haralalka et al., 2003; Dean et al., 2009; Vogt et al., 2011; Xu et al., 2016). These animal models provided evidence that the stringent response is crucial during infection, and we recently demonstrated that the stringent response of Gram-positive *S. aureus* can be effectively directly targeted *in vivo* [15] by small synthetic cationic anti-biofilm peptides (Pletzer et al., 2016).

Synthetic peptides are short (12–50 amino acids) cationic derivatives of host defense peptides that have potent broad-spectrum activities including antimicrobial, antibiofilm and immunomodulatory properties (de la Fuente-Núñez et al., 2012; Haney and Hancock, 2013). Their distinct mechanism of action against biofilms has been linked to a disruption of the stringent stress response since anti-biofilm peptides act by binding to and triggering the degradation of ppGpp. Disabling the stringent response therefore prevents intracellular accumulation of ppGpp (de la Fuente-Núñez et al., 2014), likely leading to phenotypes similar to those of stringent stress response mutants that completely lack the ability to make ppGpp.

Here, we provide new evidence describing how synthetic cationic peptides target the *Pseudomonas aeruginosa* stringent response *in vitro* and *in vivo* in a murine abscess model. *P. aeruginosa*Δ*relA*/Δ*spoT* double mutants were generated in both strain PAO1 and the clinical epidemic cystic fibrosis isolate LESB58, and these strains were complemented by chromosomal insertion of either *relA* or *spoT* under their native promoter. Using these mutants and their complements, we examined, both *in vitro* and *in vivo*, the link between the stringent stress response, virulence, and therapeutic treatment with synthetic peptides. Several virulence factors were decreased in expression in stringent response deficient mutants that caused pronounced virulence defects against human cells *in vitro*, as well as attenuated virulence in a mouse cutaneous abscess model. This also enabled targeting by synthetic cationic anti-biofilm peptides that selectively inhibited the stringent response of *P. aeruginosa in vivo*.

## Materials and Methods

### Bacterial Strains and Growth Conditions

Bacterial strains used in this study are listed in **Table 1**. Plasmids are listed in Supplementary Table S1 in the Supplementary Material. All organisms were cultured at 37°C in LB (Thermo Scientific), double Yeast Tryptone (dYT), King’s B (KB), or modified synthetic cystic fibrosis medium (MSCFM) (Palmer et al., 2007; Yeung et al., 2012). In order to enhance the expression of *P. aeruginosa* virulence factors (Bains et al., 2012; Gellatly and Hancock, 2013), iron and phosphate were reduced in MSCFM to 0.9 μM and 1 mM, respectively. Liquid cultures were shaken at 250 rpm, 37°C. Cultures harboring individual vectors were supplemented with 15 μg/ml gentamicin (Gm), 100 μg/ml ampicillin (Ap) for *Escherichia coli*, 50 μg/ml Gm for PAO1, and 500 μg/ml Gm for LESB58. Bacterial growth was monitored using a spectrophotometer at an optical density of 600 nm (OD_600_).

**Table 1:** Bacterial strains used in this study.

Strain	Relevant characteristics or genotype^a^	Reference or source
***Escherichia coli***		
XL1-Blue	recA1 endA1 gyrA96 thi-1 hsdR17 (rK- mK+) supE44 relA1 lac,[F′ proAB lacIq ZΔM15Tn10 (Tc^r^)]	Stratagene
ST18	pro thi hsdR+ Tpr Smr; chromosome::RP4-2 Tc::Mu-Km::Tn7/aaapir ΔhemA	Thoma and Schobert, 2009
Sm17λpir	Tp^r^ Sm^r^ recA, thi, pro, hsdR-M+RP4: 2-Tc:Mu: Km, aaapir phage lysogen	Simon et al., 1983
***Pseudomonas aeruginosa***		
PAO1	Laboratory wild-type strain	Hancock and Carey, 1979
PAO1.Δ*relA*	*relA* deletion mutant	This study
PAO1.Δ*relA*/Δ*spoT*	Δ*relA*/Δ*spoT* double deletion mutant	This study
PAO1.Δ*relA*/Δ*spoT* (complement *relA*)	Δ*relA*/Δ*spoT* deletion mutant chromosomally complemented with the *relA* gene including its promoter region	This study
PAO1.Δ*relA*/Δ*spoT* (complement *spoT*)	Δ*relA*/Δ*spoT* deletion mutant chromosomally complemented with the *spoT* gene including the *rpoZ*-*spoT* promoter region	This study
PAO1.(*16S*-*lux*)	Chromosomal insertion of the *16S* promoter region fused to the *luxABCDEF* reporter genes	This study
PAO1.(*relA*-*lux*)	Chromosomal insertion of the *relA* promoter region fused to the *luxABCDEF* reporter genes	This study
PAO1.(*spoT*-*lux*)	Chromosomal insertion of the *rpoZ-spoT* promoter region fused to the *luxABCDEF* reporter genes	This study
LESB58	Liverpool Epidemic Strain isolate	Cheng et al., 1996
LESB58.Δ*relA*	*relA* deletion mutant	This study
LESB58.Δ*relA*/Δ*spoT*	Δ*relA*/Δ*spoT* double deletion mutant	This study
LESB58.Δ*relA*/Δ*spoT* (complement *relA*)	Δ*relA*/Δ*spoT* deletion mutant chromosomally complemented with the *relA* geneincluding its promoter region	This study
LESB58.Δ*relA*/Δ*spoT* (complement *spoT*)	Δ*relA*/Δ*spoT* deletion mutant chromosomally complemented with the *spoT* gene including its promoter region	This study


^a^*Antibiotic resistance: Gm^r^, gentamicin; Tc^r^, tetracycline; Tp^r^, trimethoprim; Sm^r^, streptomycin; Km, kanamycin*.

### PCR Amplifications and DNA Modifications

PCR primers are listed in Supplementary Table S2 and PCR was carried out using the DreamTaq DNA polymerase (Thermo Scientific) in accordance with the manufacturer’s instructions and optimized annealing temperatures for each primer set. For PCR reactions performed with PAO1 or LESB58, bacterial cells were boiled at 98°C (1,000 rpm, 10 min) and subsequently pelleted at 13,000 rpm for 2 min. PCR reactions were supplemented with 5% dimethyl sulfoxide. For high fidelity PCR reactions, the Phusion DNA polymerase (Thermo Scientific) was used.

Restriction digestions were performed using Thermo Scientific FastDigest restriction enzymes according to the manufacturer’s instructions. All ligation reactions were carried out at room temperature using Thermo Scientific T4 DNA ligase. DNA purifications were either performed using the GeneJET PCR purification kit (Thermo Scientific) or the GeneJET Gel extraction kit (Thermo Scientific) following the manufacturer’s instructions.

### Peptide Synthesis and *In Vivo* Application

Peptides 1018 (VRLIVAVRIWRR-NH_2_) and DJK-5 (VQWRAIRVRVIR-NH_2_) were synthesized by CPC Scientific using solid-phase 9-flurenylmethoxy carbonyl (Fmoc) chemistry and purified to >95% purity using reverse-phase high-performance liquid chromatography (HPLC). The lyophilized peptide was initially resuspended in endotoxin-free water and further resuspended in saline for *in vivo* application.

### Construction of Unmarked PAO1/LESB58 Δ*relA* and Δ*relA*/Δ*spoT* Deletion Mutants

The construction of the knockout vectors was based on the protocol of Zumaquero et al. (2010) and carried out as previously described (Pletzer et al., 2014). Briefly, primers flanking the *relA* gene, relA-up-F1/relA-up-R1 and relA-down-F2/relA-down_R2 for PAO1, relA-up-F1/relA-upR1LES and relA-down-F2LES/relA-down-R2 for LESB58 were used to amplify the knockout alleles (approximately 500 bp). To amplify the *spoT* gene knockout alleles, spoT-up-F1/spoT-up-R1 and spoT-down-F2/spoT-down-R2 were used for both strains PAO1 and LESB58. R1 and F2 primers contained homologous overhang sequences. The obtained ‘up’ and ‘down’ fragments were used in an overlapping PCR reaction with primers up-F1/down-R2. Next, each fusion fragment was then sub-cloned using the Zero Blunt TOPO kit (Invitrogen Life Technologies) and verified by sequencing, before further transfer into the suicide vector pEX18Gm (Hoang et al., 1998) via *BamH*I/*Pst*I.

The generation of the unmarked stringent response deletion mutants in PAO1 and LESB58 was done step-wise, first deleting the *relA* gene (2.2 kb) and subsequently the *spoT* gene (2.1 kb), which resulted in a Δ*relA*/Δ*spoT* double mutant. The deletion method was based on the site-specific insertional mutagenesis strategy of Schweizer and Hoang (1995) and carried out as described previously (Pletzer et al., 2014). Briefly, *relA* deletion was accomplished by transferring the appropriate suicide vector into *E. coli* Sm17λpir and subsequently conjugating the construct into PAO1 and LESB58, respectively, with the helper plasmid pRK2013. Deletion of *spoT* was done by transferring the appropriate suicide vector into *E. coli* ST18 and biparental conjugation into PAO1 and LESB58. Transconjugants were selected on LB/dYT/KB agar plates containing 10% sucrose and gene deletion confirmed by locus-specific primers that bind outside the knockout alleles (relA_out1/relA_out2 and spoT_out1/spoT_out2). The obtained knockout fragments were verified by sequencing.

### Single Gene Chromosomal Complementation of PAO1/LESB58 Δ*relA*/Δ*spoT*

Promoter prediction was based on the information available from *http://www.pseudomonas.com* (Winsor et al., 2016) and the prokaryote promoter prediction tool PEPPER (de Jong et al., 2012). Therefore, primers relA-Pro_fwd/relA_rev were used to amplify *relA* (2.2 kb) including its 84-bp upstream promoter region. The amplified product was gel-purified, cloned into pUC-mini-Tn7-Gm via *Apa*I/*Spe*I, and verified by sequencing. The construct was co-electroporated with the helper plasmid pTNS3 into sucrose-prepared electrocompetent *P. aeruginosa* PAO1 and LESB58 Δ*relA*/Δ*spoT* cells, respectively. Briefly, *Pseudomonas* strains were made electrocompetent with 300 mM sucrose in three-washing steps. Next, 500 ng of each plasmid was mixed with competent cells, co-electroporated at 2.5 kV, and recovered for 3 h before plating on the appropriate selection plates. Successful integration onto the chromosome was verified with primers Tn7L/glmS_down and Tn7R/glmS_up, respectively, and subsequent sequencing of the amplified regions. With this strategy, we were able obtain a viable double mutant complemented with *relA*, reflecting a *spoT* knockout strain.

For the complementation of *spoT*, the *rpoZ*-*spoT* operon (2.5 kb) including its upstream promoter region using primers rpoZ-Pro_fwd/spoT_rev was amplified. The amplified product was gel purified, digested with *Sac*I/*Kpn*I, and subsequently cloned into the pUC-mini-Tn7 transposon vector. The cloned fragments were verified by sequencing and further transformed in *P. aeruginosa* as described above.

### Drug Susceptibility

The MICs of drugs for *P. aeruginosa* PAO1 and LESB58 were determined by the broth microdilution assay in 96-well plates (Wiegand et al., 2008) using Mueller-Hinton broth (MHB; Difco^TM^). All tests were performed in at least triplicate following the Clinical and Laboratory Standards Institute recommendations. Bacterial growth (37°C) was examined by visual inspection after 16 h (PAO1) to 48 h (LESB58) of incubation. The MIC was defined as the lowest concentration of a compound that completely prevented visible cell growth.

### Generation of the PAO1 Promoter-Bioluminescent Reporter Fusions

Transcriptional fusions between the promoter regions of *16S*, *relA*, and *spoT*, respectively, and the promoterless *luxCDABE* genes were created on plasmid pUC18T-min-Tn7T-lux (Damron et al., 2013). Briefly, the relevant upstream regions were PCR amplified either with 16S-Pro_fwd/16S-Pro_rev, relA-Pro_fwd/relA-Pro_rev, or rpoZ-Pro_fwd/rpoZ-Pro_rev, which had *BamH*I/*Pst*I restriction sites incorporated into the primer sequence, and then further subcloned into pTOPO-pCR-BluntII. Amplified regions were subsequently verified by sequencing. Next, the *P1* integron promoter was excised from pUCP18T-miniTN7-lux-Gm using *BamH*I/*Pst*I restriction enzymes and replaced with the amplified promoter regions. Obtained plasmids were transferred onto the PAO1 chromosome as described above.

### Cell Culture and Cytotoxicity Assay

The human bronchial epithelial cell line 16HBE14o- (HBE; a gift from D. Gruenert, University of California, San Francisco) was maintained in minimal essential medium (MEM, Thermo Fisher Scientific) supplemented with 10% fetal bovine serum (Thermo Fisher Scientific), L-glutamine (2 mM, Thermo Fisher Scientific) and penicillin/streptomycin (100 U/ml, Thermo Fisher Scientific) at 37°C in 5% CO_2_.

After overnight growth in MSCFM medium (20 h PAO1; 22 h LESB58), bacterial cells were pelleted at 5000 × *g* (15 min) and bacterial supernatants (75 μl PAO1, 150 μl LESB58) were used to treat confluent monolayers of HBE cells (8 × 10^4^ cells/well) for 1 h at 37°C (200 μl total reaction volume with MSCFM). HBE cells treated with Triton X-100 (2% v/v) served as a positive control and MSCFM-treated HBE cells as a negative control. Lactate dehydrogenase (LDH) release into the cell culture supernatant was determined by using a cytotoxicity detection kit (Roche) according to the manufacturer’s instructions.

### Hemolysis Assay

Human red blood cells (RBCs) from healthy volunteer donors were used to measure the release of hemoglobin after incubation with *P. aeruginosa* supernatants. Fresh blood was collected in sodium heparin blood collection tubes (BD Vacutainer^®^, VWR) and RBCs subsequently isolated by centrifugation (500 × *g*, 10 min). RBCs were washed three times (500 × *g*, 10 min) using phosphate buffered saline (PBS, Thermo Fisher Scientific) and subsequently resuspended and stored in an equal volume of Alsever’s solution (Sigma–Aldrich) at 4°C for a maximum of 4 weeks.

After overnight growth of *P. aeruginosa* (20 h PAO1; 22 h LESB58), bacterial cells were pelleted at 5000 × *g* (15 min), and the supernatants (50 μl PAO1, 100 μl LESB58) further incubated with 1% RBC (washed three times [500 × *g*, 10 min] with MSCFM prior to usage) in a 200 μl reaction volume with MSCFM. Triton X-100 (2% v/v, Sigma–Aldrich)-treated RBC served as a positive control and MSCFM-treated RBC as a negative control. After incubation for 1 h at 37°C, RBCs were pelleted at 500 × *g* (10 min), and the hemoglobin content of the supernatants measured at 450 nm (reference 630 nm) using a microplate reader. Hemolysis percentage was calculated by (ΔOD_sample_ – ΔOD_negative control_)/(ΔOD_positive control_ – ΔOD_negative control_) × 100.

### RNA Isolation and Quantitative Real-time (qRT)-PCR

To further investigate their hemolytic activity, PAO1, LESB58, and their corresponding Δ*relA*/Δ*spoT* double mutants were grown on KB plates supplemented with 10% blood. For PAO1, 2-day grown colonies, for LESB58, 3-day grown colonies were scraped from the blood-agar plates, resuspended in RNAprotect Bacteria Reagent (QIAGEN) and further harvested by centrifugation (13,000 rpm, 2 min). Total RNA was isolated using the RNeasy Mini Kit (QIAGEN) following the manufacturer’s instructions. The obtained RNA was DNAse-treated (Ambion/Life Technologies) and subsequently quantified using a Nanodrop ND-2000 spectrophotometer (Thermo Fischer Scientific) and RNA integrity determined by agarose gel electrophoresis.

High quality RNA was reverse transcribed and amplified with a Roche LightCycler 96 instrument, in combination with the qScript^TM^ One-Step SYBR^®^ Green qRT-PCR Kit (QuantaBio) according to the manufacturer’s protocol. Template RNA (5 ng/sample) was used in a standard 25 μl qRT-PCR reaction with specific primers (Supplementary Table S2). Each sample was analyzed for gene expression in at least triplicate. Quantification of mRNA transcripts was performed by the comparative C_t_ method (Schmittgen and Livak, 2008) using *rpoD* as normalizer.

### Ethics Statement

Animal experiments were performed in accordance with The Canadian Council on Animal Care (CCAC) guidelines and were approved by the University of British Columbia Animal Care Committee (certificate number A14-0363). Mice used in this study were female outbred CD-1. All animals were purchased from Charles River Laboratories (Wilmington, MA, United States), were 7 weeks of age, and weighed about 25 ± 3 g at the time of the experiments. 1–3% isoflurane was used to anesthetize the mice. Mice were euthanized with carbon dioxide.

Donated human blood was collected from healthy, consenting volunteers using protocols approved by UBCs Clinical Research Ethics Board. A written consent was obtained from all blood donors and the samples subsequently anonymized.

### Cutaneous Mouse Infection Model

The abscess infection model was performed as described earlier (Pletzer et al., 2017). Initially, experiments were performed using an inocula of 5 × 10^7^ organisms for PAO1 and LESB58. However, this inoculum caused 20–30% mortality when PAO1 was used. Since we continued to work with this strain, we performed pathogen-dose experiments and found that we could reduce the inoculum to 1 × 10^7^ organisms, which was still responsible for 15% mortality, but significantly reduced fluctuations in abscess sizes. For the experiment, the fur on the backs of the mice was removed by shaving and application of chemical depilatories. *P. aeruginosa* PAO1 and LESB58, respectively, were grown to an OD_600_ of 1.0 in dYT broth. Prior to injection, bacterial cells were washed twice with sterile PBS and adjusted to 1–5 × 10^7^ CFU/ml for PAO1 and 5 × 10^7^ CFU/ml for LESB58. A 50 μl bacterial suspension was injected into the right side of the dorsum. All utilized peptides were tested for skin toxicity prior to efficacy testing. Concentrations used were 10 mg/kg for 1018 and 3 mg/kg for DJK-5. Peptides or saline (50 μl) were directly injected subcutaneously into the infected area [intra-abscess (IA) injection] at 1 h post-infection. The progression of the disease/infection was monitored daily and abscess lesion sizes (visible dermonecrosis) on day 3 measured using a caliper. Swelling/inflammation was not considered in the measurements. Skin abscesses were excised (including all accumulated pus), homogenized in sterile PBS using a Mini-Beadbeater-96 (Biospec products) for 5 min, and bacterial counts determined by serial dilution. Experiments were performed at least three times independently with 2–4 animals per group.

### Tracking Promoter Activity with Bioluminescence *In Vivo*

The PAO1 *16S* gene promoter, fused to a bioluminescence gene, was used to follow disease progress in real-time. Additionally, the *16S* promoter fusion served as control for peptide induction studies since it showed constant expression independent of the applied treatment. For induction studies, the *relA* as well as *rpoZ*-*spoT* promoter fused to bioluminescence genes were used. Peptides were injected into the abscess 1 h post-infection as described above and promoter activity measured 1, 24, 48, and 72 h post-treatment. Bioluminescence images were acquired (60 s exposure, medium binning) using the IVIS Lumina system (Perkin Elmer) and analyzed using Living Image software.

### Tracking of Reactive Oxygen and Nitrogen Species (ROS/RNS) and Neutrophils *In Vivo*

Mice were infected with *P. aeruginosa* LESB58 and subsequently treated with saline (control) or peptide 1018 as described above. To detect the production of ROS/RNS the chemiluminescence probe L-012 (Kielland et al., 2009) was used. To measure and visualize neutrophil chemotaxis and activation, the neutrophil-specific fluorescent NIR dye (Kerafast) was used. Both were applied as described earlier (Pletzer et al., 2017). Representative images, taken at 3, 24, 48, or 72 h, were acquired using the IVIS Lumina system (luminescence: 60 s exposure, medium binning; fluorescence: excitation 745 nm, emission 800 nm) and analyzed using Living Image software. Fluorescent images were subjected to adaptive background subtraction and the fluorescence emission normalized to the incident excitation intensity (radiance of the subject/illumination intensity).

### Statistical Analysis

Statistical evaluations were performed using GraphPad Prism 7.0 (GraphPad Software, La Jolla, CA, United States). *p*-values were calculated using one-way ANOVA, Kruskal–Wallis multiple-comparison test followed by the Dunn procedure. Data was considered significant when *p*-values were below 0.05 or 0.01 as indicated.

## Results

### Construction and Complementation of the *P. aeruginosa* PAO1 and LESB58 Δ*relA*/Δ*spoT* Mutants

To enable the study of the stringent response, we created single and double mutants of *relA* and *spoT* in the two *P. aeruginosa* strains PAO1 and LESB58. Several attempts failed to knock out *spoT*, the ppGpp synthase/hydrolase. Moreover, preliminary experiments to overexpress *relA* in a PAO1 wild-type showed that a strong induction of the gene is lethal to the strain, indicating that an imbalance of *relA* expression affect growth. Hence, we concluded that a *spoT*-deficient strain was not viable in either *P. aeruginosa* strain, most likely due to the accumulation of ppGpp inside the cell due to the activity of the ppGpp synthase, RelA. Similar observations have been reported in *P. aeruginosa* (Vogt et al., 2011) and *E. coli* (Xiao et al., 1991). However, since other non-specific hydrolases have recently been discovered that are capable of degrading ppGpp, such as the nudix pyrophosphatase in *Thermus thermophilus* (Ooga et al., 2009), we hypothesized that *Pseudomonas* might be able to survive in the presence of low concentrations of ppGpp. To test this concept, we created a Δ*relA*/Δ*spoT* double knockout strain by successive deletion of the *relA* gene followed by *spoT* to obtain ppGpp-deficient mutants in both PAO1 and LESB58.

To complement the Δ*relA*/Δ*spoT* double mutant, we used single chromosomal insertions of either the *relA* and *spoT* gene with their corresponding upstream promoter sequences. The prediction tool PEPPER (de Jong et al., 2012) and the information provided at *http://www.pseudomonas.com* (JBrowse) (Winsor et al., 2016) were used to identify the corresponding promoter regions. Transcription start site prediction indicated that the *relA* gene from both *P. aeruginosa* PAO1 and LESB58 was transcribed from two distinct promoters, as previously described for *E. coli* (Nakagawa et al., 2006). In *E. coli*, the promoter directly upstream of the *relA* gene was found to be constitutively active, while the second promoter, found further upstream, was induced during the transition into stationary phase (Nakagawa et al., 2006). To test our hypothesis that *P. aeruginosa* might survive in the presence of low levels of ppGpp, we used the directly adjacent promoter region upstream of the *relA* gene to complement the Δ*relA*/Δ*spoT* mutant and found that cells with this construct were indeed viable and thus partly resembled a *spoT* knockout in both *P. aeruginosa* PAO1 and LESB58. To complement the Δ*relA*/Δ*spoT* double mutant with the *spoT* gene, the *rpoZ*-*spoT* operon including its upstream promoter region was chromosomally inserted, and the corresponding mutant partially resembled a *relA* knockout phenotype (**Table 1**).

### *P. aeruginosa* Stringent Response Mutants Exhibited Attenuated Virulence against Human Bronchial Epithelial Cells

**FIGURE 1:**
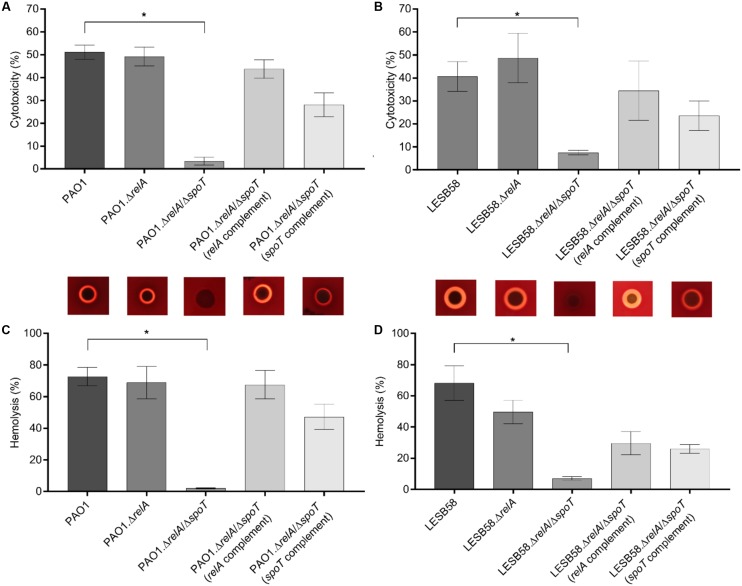
Cytotoxicity and hemolysis of *Pseudomonas aeruginosa* PAO1/LESB58, stringent response mutants, and complemented strains. The release of lactate dehydrogenase, from human bronchial epithelial cells incubated for 1 h with supernatants of the indicated strains, into culture supernatants was measured. Hemolytic activity of the strains was determined by either incubating red blood cells with supernatants collected from overnight cultures, or by plating the bacteria onto blood agar plates. **(A)** Cytotoxicity of PAO1 strains. **(B)** Cytotoxicity of LESB58 strains. **(C)** Hemolysis of PAO1 strains on blood agar plates (top) and assessment of hemoglobin release into culture supernatants (bottom). **(D)** Hemolysis of LESB58 strains on blood agar plates (top) and assessment of hemoglobin release into culture supernatants (bottom). **(A–D)** Statistical analysis was performed using one-way ANOVA, Kruskal–Wallis test with Dunn’s correction. The asterisk indicates a significant difference to the wild-type (*p* ≤ 0.01). Error bars, mean ± SEM.

Since the stringent response has been implicated in the regulation of bacterial virulence (Dalebroux et al., 2010; Vogt et al., 2011), we tested whether the *P. aeruginosa* PAO1 and LESB58 stringent response mutants secreted less cell-damaging virulence factors when compared to the parent strains. Therefore, human bronchial epithelial (HBE) cells were incubated with overnight-grown bacterial supernatants and the release of the cytosolic enzyme LDH from damaged HBE cells was quantified. Our data showed that LESB58 was about 10% less cytotoxic when compared to PAO1 in this experiment (**Figures 1A,B**).

As anticipated, cytotoxicity was reduced in both *P. aeruginosa* Δ*relA*/Δ*spoT* double mutant strains (PAO1 and LESB58) when compared to their wild-type parents. The PAO1 Δ*relA*/Δ*spoT* mutant showed about 48% less cytotoxicity while the LESB58 Δ*relA*/Δ*spoT* mutant showed a reduction of 33%. Single gene complementation of the double mutant with either *relA* or *spoT* increased cytotoxicity levels. Deletion of only *relA* had no significant impact on cytotoxicity in either in the PAO1 or LESB58 backgrounds (**Figures 1A,B**).

### PAO1/LESB58 Stringent Response Mutants Showed Reduced Hemolytic Activity

Due to the observed decreased cytotoxicity of the Δ*relA*/Δ*spoT* strains, we further evaluated the release of hemoglobin from RBCs when incubated with overnight-grown bacterial supernatants. *P. aeruginosa* PAO1 and LESB58 showed similar hemolytic activity of approximately 70%. Interestingly, the PAO1 Δ*relA*/Δ*spoT* double mutant showed almost complete reduction in hemolysis (**Figure 1C**), while a Δ*relA* mutant as well as the *relA* and *spoT* complemented double mutant strains showed similar hemolysis levels as the wild-type. In LESB58, a reduction in hemolysis was observed for all tested mutants and complemented strains, with a significant reduction (*p* < 0.01) in the Δ*relA*/Δ*spoT* double mutant (approximately 60%) compared to the wild-type (**Figure 1D**).

To further elucidate the mechanism behind the decreased hemolytic activity of the stringent response double mutant, we isolated bacterial RNA from cells grown on blood agar plates. As with the previous supernatant assay, PAO1 Δ*relA*/Δ*spoT* showed no hemolytic activity, while LESB58 Δ*relA*/Δ*spoT* was still able to lyse some RBCs (**Figures 1C,D**, top panel). We compared the Δ*relA*/Δ*spoT* mutant strains to their corresponding wild-type parents and examined gene expression for various virulence-associated factors by qRT-PCR. Interestingly, more virulence-associated genes were dysregulated in the PAO1 stringent response mutant than in LESB58 mutant (**Table 2**). In particular the *aprA* gene, encoding an alkaline protease with hemolytic activity (Hong and Ghebrehiwet, 1992), was about 11.2-fold down-regulated in PAO1 Δ*relA*/Δ*spoT* and 4.8-fold down-regulated in LESB58 Δ*relA*/Δ*spoT*, which might explain the loss of cytolytic activity in the stringent response double mutants. Differential hemolytic activity in the two genetic backgrounds might have resulted from the different fold decreases in expression of AprA or from other unique differences. Thus for strain PAO1 there was a >3-fold reduction in expression for genes encoding the secreted hemolysin, phospholipase C (*plcB*), a phenazine biosynthesis gene (*phzB1*), rhamnosyltransferase chain B (*rhlB*), and the galactose binding lectin A gene (*pa1L*), as well as a >2-fold increased expression of genes encoding the Type III secretion system (*exsA*, *pscF*, *esxD*, and *pscI*) and a siderophore transporter (*pvdE*). Conversely, LESB58 Δ*relA*/Δ*spoT* showed reduced expression, by more than twofold, of the genes encoding pyoverdine expression, *pvdE*, and the type II secretion substrates lipase A (*lipA*) and exotoxin A (*toxA*).

**Table 2:** Relative fold-changes of *P. aeruginosa* PAO1 Δ*relA*/Δ*spoT* and LESB58 Δ*relA*/Δ*spoT* mRNA expression compared to their respective wild-type levels of expression.

Gene	Locus (PAO1/LESB58)	Virulence factor/description	Fold change in Δ*relA*/Δ*spoT*	
			PAO1	LESB58
*aprA*	PA1249/PALES_40631	Alkaline protease, Type I secretion	-11.2	-4.8
*lasB*	PA3724/PALES_12581	Elastase type II secretion system substrate	-10.6	1.8
*pa1L*	PA2570/PALES_27241	Galactose binding lectin A	-10.2	-1.7
*phzB1*	PA4211/PALES_07161	Phenanzine biosynthesis type II secretion	-4.3	1.2
*rhlB*	PA3478/PALES_15341	Rhamnolipid rhamnosyltransferase chain B	-3.3	-1.5
*plcB*	PA0026/PALES_00251	Phospholipase C, Type II secretion	-3.0	-1.1
*lipA*	PA3996/PALES_22021	Lipase A type II secretion system substrate	-1.4	-2.7
*exoS*	PA3841/PALES_11331	Exoenzyme S type III secretion substrate	-1.4	n.d.
*pscC*	PA1716/PALES_36131	Type III secretion outer membrane protein	1.3	1.2
*pchB*	PA4230/PALES_06971	Pyochelin siderophore biosynthesis	1.4	-1.5
*toxA*	PA1148/PALES_41711	Exotoxin A type II secretion system substrate	1.8	-2.4
*pscI*	PA1722/PALES_36071	Type III secretion export protein	2.1	n.d.
*pvdE*	PA2397/PALES_28991	Pyoverdine siderophore ABC transporter	2.1	-2.0
*exsD*	PA1714/PALES_36151	Type III secretion system negative regulator	3.8	1.6
*pscF*	PA1719/PALES_36101	Type III secretion needle	4.3	1.3
*exsA*	PA1713/PALES_36161	Type III secretion system transcriptional activator	4.6	1.2


*Strains were grown on KB agar plates supplemented with 10% blood for 2 (PAO1) and 3 (LESB58) days, respectively. Transcript abundance was determined by qRT-PCR and represent the means of at least three biological replicates. n.d., not determined*.

### The Stringent Response Was Required for Cutaneous Abscess Tissue Necrosis in Mice

Bacteria found in wounds or chronic skin infections are thought to experience high levels of stress due to the rapid release of reactive oxygen species (i.e., oxidative burst) by host-related defense mechanisms such as macrophages and neutrophils (Slauch, 2011; Dhall et al., 2014). Additional limitations within a wound (e.g., nutrient or iron deprivation due to the high density of organisms) may also contribute to stress-related responses. We hypothesized that these factors are under the control of the bacterial stringent stress response. Therefore, we tested in a murine cutaneous infection model of high-density infections, the effects of inoculation of 5 × 10^7^ CFU of *P. aeruginosa* strains PAO1 and LESB58 wild-type, Δ*relA*, and Δ*relA*/Δ*spoT* mutants with their corresponding complemented strains. These studies investigated their ability to cause abscesses and/or tissue necrosis, the bacterial burden 3 days post-infection, and the survival rate of the infected mice.

**FIGURE 2:**
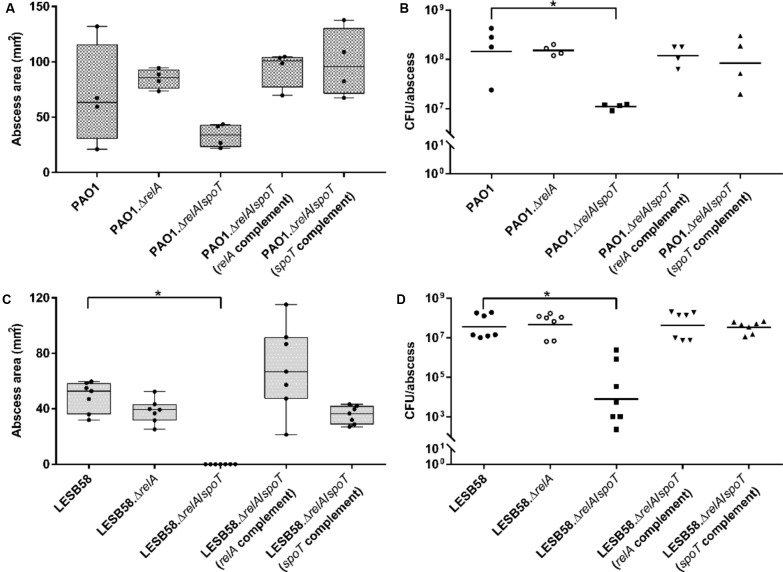
*Pseudomonas aeruginosa* stringent response strains in a cutaneous mouse infection model. CD-1 mice were subcutaneously infected with either PAO1 or LESB58, their corresponding stringent response mutants Δ*relA* and Δ*relA*/Δ*spoT*, as well as the Δ*relA*/Δ*spoT* complemented strains. A high bacterial density (5 × 10^7^ CFU) was used. Lesion sizes (Box and whiskers plot) and CFU counts (with geometric mean) were determined 3 days post-infection. **(A)** PAO1 dermonecrosis measurements. **(B)** PAO1 CFU counts/abscess. **(C)** LESB58 dermonecrosis measurements. **(D)** LESB58 CFU counts/abscess. **(A–D)** All experiments were done at least three times independently with 2–4 mice/group. Statistical analysis was performed using one-way ANOVA, Kruskal–Wallis test with Dunn’s correction. The asterisk indicates a significant difference to the wild-type (*p* < 0.05).

The PAO1 Δ*relA*/Δ*spoT* showed visible, but not statistically significant, decreases in abscess sizes of 44% when compared to the wild-type (**Figure 2A**). However, the *relA* and *spoT* complemented Δ*relA*/Δ*spoT* double mutant strains formed significantly greater abscesses by >2-fold compared to the double mutant. PAO1 Δ*relA*/Δ*spoT* further showed a significant 11.6-fold lower recovery of viable cells (CFU) 3 days post-infection, while the *relA*-deficient strain as well as the complemented double mutants showed similar bacterial counts, comparable to the wild-type (**Figure 2B**).

In the LESB58 background strain, the Δ*relA*/Δ*spoT* double mutant was unable to cause dermonecrosis while genetic complementation with either *relA* or *spoT* restored the ability to cause tissue damage (**Figure 2C**). Deletion of only the *relA* synthase showed a similar phenotype as the wild-type. Interestingly, although the double mutant showed no visible tissue dermonecrosis, bacterial cells were still recovered from the injection site 3 days post-infection. However, the LESB58 Δ*relA*/Δ*spoT* double mutant showed 10^4^-fold less CFU when compared to the wild-type (**Figure 2D**). As previously demonstrated (Pletzer et al., 2017), LESB58 showed no lethality in this model.

**FIGURE 3:**
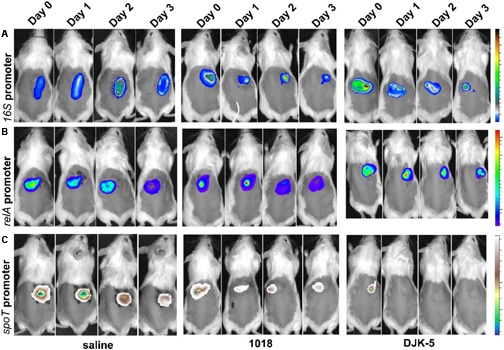
*In vivo* tracking of *P. aeruginosa* PAO1 *16S*, *relA*, and *spoT* promoter activity. The infection progress was monitored at the indicated time points. Female CD-1 mice were injected with a high bacterial number (5 × 10^7^ CFU PAO1) and treated with either saline (control, left panel) or peptides 1018 (middle panel) and DJK-5 (right panel) 1 h post-infection. **(A)** *16S* promoter activity as induction/suppression control for peptide treatment. Radiance color scale from 0.5 to 2.5 × 10^9^. **(B)** *relA* promoter activity. Radiance color scale from 0.5 to 4.5 × 10^7^. **(C)** *spoT* promoter activity. Radiance color scale from 0.5 to 4.5 × 10^7^. **(A–C)** All experiments were done at least twice with three mice/group.

Since environmental factors could play a role in the stimulation of ppGpp production, we investigated the expression of *relA* and *spoT* using a bioluminescent reporter fusion to track the infection progress *in vivo*. Previously, we demonstrated that *in vivo* tracking of bacterial infection using bioluminescence was achievable with LESB58 in this cutaneous abscess model (Pletzer et al., 2017). Here, we created chromosomal promoter–reporter fusions in PAO1 and tracked the infection for 3 days. As shown in **Figure 3**, the *16S* promoter–reporter fusion served as an infection control and was trackable in saline-treated mice with constant expression over 3 days (**Figure 3A**, left panel). Stringent response gene promoter fusions of *relA* and *spoT* to the bioluminescence reporter when administered to saline-treated mice, resulted in luminescence that was detectable throughout the experiment (**Figures 3B,C**, left panel). Notably, the expression intensity of the *16S* construct was about 100-fold higher when compared to *relA* or *spoT* expression and although the signal intensity decreased over time it did not influence the experiment.

### Targeting the Stringent Response with Peptides 1018 and DJK-5 *In Vivo*

**FIGURE 4:**
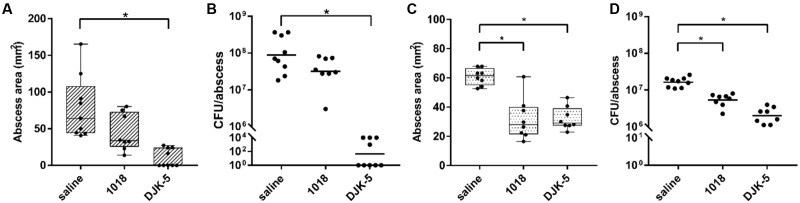
Therapeutic treatment with synthetic peptides of PAO1 and LESB58 infected mice. CD-1 mice were subcutaneously infected with 5 × 10^7^ CFU *P. aeruginosa* PAO1 or LESB58 and treated intra-abscess with either saline (control) or synthetic peptides 1018 (10 mg/kg) and DJK-5 (3 mg/kg) 1 h post-infection. Lesion sizes and CFU counts were determined 3 days post-infection. **(A)** Box and whiskers plot of dermonecrosis measurements for PAO1 infections. **(B)** CFU counts/abscess with geometric mean for PAO1 infections. **(C)** Box and whiskers plot of dermonecrosis measurements for LESB58 infections. **(D)** CFU counts/abscess with geometric mean for LESB58 infections. **(A–D)** All experiments were done at least three times with 2–4 mice/group. Statistical analysis was performed using one-way ANOVA, Kruskal–Wallis test with Dunn’s correction. The asterisk indicates significant differences to the wild-type (*p* < 0.05).

Interestingly, bacterial abscess infections can be treated with synthetic anti-biofilm peptides (Mansour et al., 2016), although biofilm formation and abscess infections are not necessarily related to each other. However, it was previously shown that these peptides target the stringent response *in vitro* (de la Fuente-Núñez et al., 2014) and that this led to control of the formation of dermonecrotic lesions *in vivo* for Gram-positive *S. aureus* cutaneous infections (Mansour et al., 2016). Although there was a therapeutic effect of DJK-5 against *P. aeruginosa* infections [15], it was not confirmed to be linked with the stringent response. Here, given the impact of the stringent response on tissue dermonecrosis formation and survival within the abscess (**Figure 2**), we further investigated the treatment of *P. aeruginosa* abscess infections with anti-biofilm peptides 1018 and DJK-5, which both target the stringent response *in vitro* (de la Fuente-Núñez et al., 2014). Their respective MIC value against PAO1 in MHB was 25 μg/ml for 1018 and 12.5 μg/ml for DJK-5 and for LESB58 64 μg/ml for 1018 and DJK-5. We separately administered peptides 1018 (10 mg/kg) and DJK-5 (3 mg/kg) directly into the abscess 1 h after the infection was initiated, and found that the peptides significantly reduced tissue dermonecrosis by ∼50% for PAO1 (DJK-5 only; although the appearance of 1018-treated lesions indicate less severe lesions) and LESB58 (**Figures 4A,C**). The number of recovered bacteria varied between the strains. While single dose treatment with 1018 reduced the bacterial load by approximately 2.8-fold in PAO1 (non-significantly) and 3.2-fold in LESB58 (*p* < 0.05), DJK-5 almost completely eradicated bacteria (>10^6^-fold reduction) in the case of strain PAO1 and significantly reduced bacteria numbers by about 10-fold for strain LESB58 (**Figures 4B,D**) at 3 days post-infection. Interestingly, 1018, a profound immune modulator (Mansour et al., 2015), did not significantly affect ROS/RNS levels or the influx of neutrophils (Supplementary Figure S1) that were evident during *P. aeruginosa* abscess formation (Pletzer et al., 2017).

**FIGURE 5:**
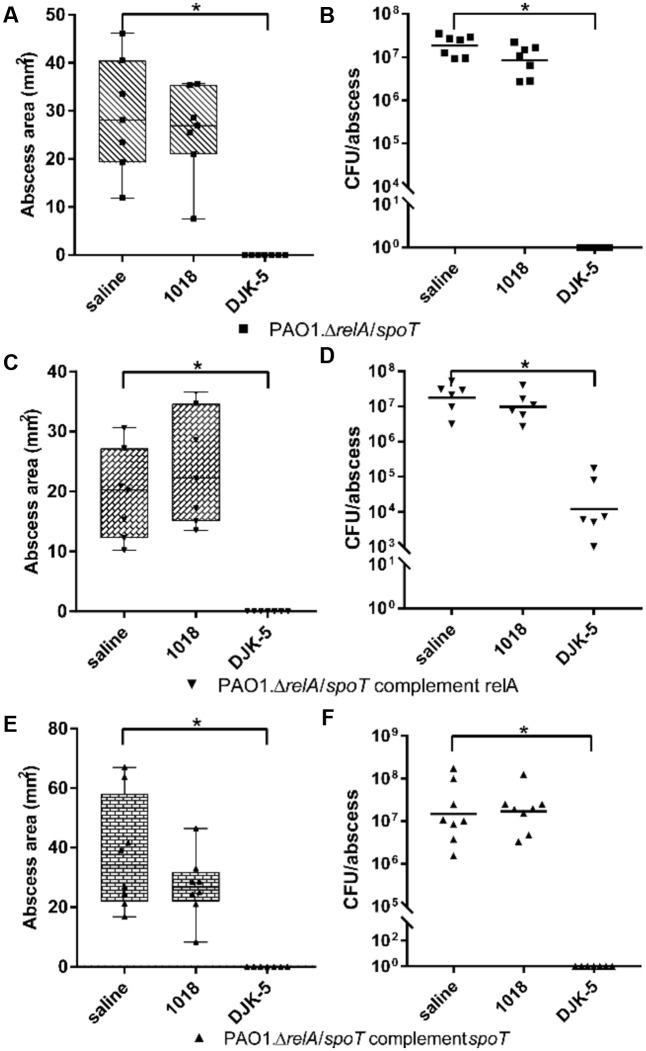
Therapeutic treatment of PAO1 stringent response mutants and complemented strains with synthetic peptides. CD-1 mice were subcutaneously infected with 1 × 10^7^ CFU *P. aeruginosa* PAO1, Δ*relA*/Δ*spoT*, and corresponding complemented strains. Intra-abscess administration of either saline (control) or synthetic peptides 1018 (10 mg/kg) and DJK-5 (3 mg/kg) was done 1 h post-infection. Lesion sizes (Box and whiskers plots) and CFU counts (with geometric mean) were determined 3 days post-infection. **(A)** PAO1.Δ*relA*/Δ*spoT* dermonecrosis measurements. **(B)** PAO1.Δ*relA*/Δ*spoT* CFU counts/abscess. **(C)** PAO1.Δ*relA*/Δ*spoT* complemented with *relA* dermonecrosis measurements. **(D)** PAO1.Δ*relA*/Δ*spoT* complemented with *relA* CFU counts/abscess. **(E)** PAO1.Δ*relA*/Δ*spoT* complemented with *spoT* dermonecrosis measurements. **(F)** PAO1.Δ*relA*/Δ*spoT* complemented with *spoT* CFU counts/abscess. **(A–F)** All experiments were done at least three times with 2–4 mice/group. Statistical analysis was performed using one-way ANOVA, Kruskal–Wallis test with Dunn’s correction. The asterisk indicates a significant difference to the wild-type (*p* < 0.05). Data points for mice with no visible abscess or no recovered CFU were set to 1 to perform statistical analysis.

To further elucidate the effect of the peptides on the stringent response *in vivo*, we used the PAO1 Δ*relA*/Δ*spoT* double mutant, and Δ*relA*/Δ*spoT* complemented with *relA* or *spoT* (**Figure 5**). Interestingly, peptide 1018 was ineffective vs. mice treated with the Δ*relA*/Δ*spoT* double mutant in that there was no visible reduction in abscess lesion sizes and an insignificant reduction of recovered bacteria (**Figures 5A,B**). However, consistent with its greater potency, treatment with DJK-5 completely eradicated the infections. The Δ*relA*/Δ*spoT* strain complemented with *relA*, resembling a *spoT* knockout, showed a large inconsistency in terms of tissue necrosis formation and the amount of recovered CFU (**Figures 5C,D**). Nevertheless, when we treated the infection with peptides, we found that 1018-treatment showed no significant effect. Intriguingly, DJK-5-treated infections still completely suppressed abscess formation (**Figure 5C**), but did not completely clear the bacteria from the injection site (**Figure 5D**), with about 1000-fold less bacteria recovered cf. the saline-treated control groups. In contrast complementation with *spoT* showed no significant differences compared to the Δ*relA*/Δ*spoT* double mutant (**Figures 5E,F**).

To understand the mechanisms involved, we tested the *in vivo* influence of the synthetic peptides 1018 and DJK-5 in induction or repression of the expression for ppGpp synthesis genes. Based on light production from the reporter *lux* operon, treatment of the *spoT*-reporter construct treated with 1018 led to reduced emitted light (decreased i*n vivo* expression) by approximately twofold at 24 h post-infection (**Figure 3C**). Even more striking was the finding that DJK-5 completely suppressed *spoT* promoter activity within 24 h (**Figure 3C**). There was, however, little change in expression of the *relA* gene (**Figure 3B**), or, as a control, the *16S* rRNA (**Figure 3A**).

Taking into account all experiments performed (with 15–24 total animals per strain), the PAO1 wild-type strain was moderately virulent in the cutaneous infection model (15% mortality), while the PAO1 Δ*relA*/Δ*spoT* showed about 12% mortality, the *relA* complementation 7% mortality and *spoT* complementation 0% mortality. These variations in mortality rates were not significantly different (as determined by Fisher’s exact test). The direct administration of peptides into abscesses of PAO1-infected mice led to 100% survival of all mice.

## Discussion

Adaptation to changing environmental conditions such as those encountered during the establishment of an infection inside a host are hallmarks of many bacteria. A rapid response to cope with limiting factors, such as fluctuations in nutrient availability and other stressors such as host defense mechanisms, can be achieved by various mechanisms including the stringent stress response. The results presented here are consistent with and extend literature studies indicating that the stringent response and its second messenger molecule ppGpp are important in the regulation of adaptive responses and bacterial virulence (Potrykus and Cashel, 2008; Dalebroux et al., 2010), as well as persistence and survival during host invasion and antibiotic resistance (Hauryliuk et al., 2015).

Initially, we found that the deletion of only the ppGpp hydrolase SpoT was lethal in *P. aeruginosa*, in accordance with other studies (Vogt et al., 2011; Manuel et al., 2012). However, we could partly complement a Δ*relA*/Δ*spoT* double mutant with a chromosomal insertion of *relA* and its constitutive promoter, which created the equivalent of a viable *spoT* knockout. We propose that this might be due to the weak expression from the native *relA* promoter that prevented excessive ppGpp production and the possibility that non-specific hydrolases might be able to degrade the more modest levels of ppGpp produced in this background. The enzymes other than SpoT that appear to influence ppGpp levels in bacteria to date, are the phosphohydrolase MazG in *E. coli* (Gross et al., 2006) and the nudix pyrophosphatase Ndx8 in *Thermus thermophilus* (Ooga et al., 2009). However, further studies are needed to explore whether such non-specific hydrolases exist in *P. aeruginosa*.

*Pseudomonas aeruginosa* relies on a suite of secreted toxins and virulence factors (including phospholipase C, exotoxin A and Type III secreted exotoxins, rhamnolipids, siderophores, the elastase and alkaline protease, and pyocyanin) to colonize its host and establish an infection (van Delden, 2004; van ’t Wout et al., 2015). Toxin production and secretion during host-microbe interactions causes distinct macroscopic pathological changes including anemia, tissue necrosis, and neural damage (Sadikot et al., 2005). One major weapon involved in cytotoxicity and acute infections in *P. aeruginosa* is the type III secretion system (Hauser, 2009) that secretes effector proteins to help evading the host tissue (Filloux, 2011). In addition our previous studies indicated that Type III secretion was essential for strain PA14 dermonecrosis (Pletzer et al., 2017). We thus hypothesized that this system might be dysregulated in stringent response mutants that showed reduced cytotoxicity against HBE cells and reduced hemolytic activity against human RBCs (**Figure 1**). After analyzing the expression of various virulence-associated factors in wild-type and the stringent response mutants (**Table 2**), we found that Type III secretion was upregulated. Conversely type-I-secreted alkaline protease AprA, a zinc metalloprotease, was the only enzyme down-regulated in both stringent response mutants of PAO1 and LESB58, respectively, and might therefore be a major contributor to the lysis of erythrocytes. AprA has been shown to aid *P. aeruginosa* survival during lung infection (Kim et al., 2006) and has also been connected to the blockage/degradation of complement proteins that otherwise rapidly detect and promote killing of Gram-negative bacteria (Laarman et al., 2012).

Stringent stress response double mutants exhibited attenuated virulence during PAO1 and LESB58 infections, respectively, with no visible dermonecrosis in LESB58. This could be a result of increased bacterial clearance or a reduced ability to adapt to and grow under the stressful conditions in the abscess. Indeed, our data showed that bacterial loads inside the abscess tissue of mice infected with the double mutant were significantly lower than in mice infected with the wild-type or complemented strains (**Figure 2**). This is partly consistent with results showing a double mutant was essentially avirulent in an acute *P. aeruginosa* pulmonary infection model (Xu et al., 2016). On the other hand, reduced virulence might also be connected to a down-regulation of the AprA protease in stringent response mutants, which might otherwise have adverse effects during host colonization. Moreover, Vogt et al. (2011) speculated that a Δ*relA*/Δ*spoT* double mutant would be more susceptible to a variety of different stresses during host interaction, which could be responsible for a reduced ability to survive attacks mediated by the host immune system. We have previously shown that reactive oxygen species are activated during infection in the cutaneous abscess model (Pletzer et al., 2017), and these could conceivably account for increased killing of the double mutant in this model.

Apart from the investigation of the role of the stringent response in virulence during a high-bacterial-density cutaneous abscess infection, we also examined the possibility of specifically targeting the stringent response *in vivo*. In this context, Wexselblatt et al. (2012) identified a stringent response inhibitor, Relacin, that directly interacted with and completely inhibited purified *E. coli* RelA enzyme. However, Relacin only reduced ppGpp production in Gram-positive *Bacillus subtilis* cells, and had no effect on *E. coli* cells, most likely due to its inability to penetrate the Gram-negative bacterial cell wall (Wexselblatt et al., 2012). Very recently, synthetic ppGpp analogs were investigated and demonstrated a down-regulation of ppGpp concentrations in *Mycobacterium smegmatis* (Syal et al., 2017). Conversely, we showed that the anti-biofilm peptides 1018 and DJK-5 were able to target and stimulate degradation of ppGpp in Gram-negative *P. aeruginosa* and Gram-positive *S. aureus* (de la Fuente-Núñez et al., 2014, 2015). Thus, to our knowledge, only the peptides 1018 and DJK-5 have been shown to provide a possible therapeutic approach against Gram-positive and Gram-negative pathogens in vertebrate infection models (Achtman et al., 2012; Mansour et al., 2016). Recently, the observation that 1018 targets the cellular stress response had been challenged (Andresen et al., 2016). However, the methodological procedures described by Andresen et al. (2016) have serious issues including the use of microtiter plate adherence assays and crystal violet staining (which does not discriminate between live cells and bacterial debris) to assess biofilm formation. Crystal violet experiments show large variations and are quite poor for accurately assessing anti-biofilm activity, especially when set up as a MIC experiment where peptides are added to planktonic cells. Furthermore, Andresen et al. (2016) tested the conditional essentiality of ppGpp on planktonic *E. coli* cells rather than biofilm cells and they tested the effects of 1018 on bacterial survival on a ppGpp-deficient strain under non-stressed conditions. Ultimately, no studies were included to demonstrate the effects of 1018 on intracellular ppGpp as previously demonstrated (de la Fuente-Núñez et al., 2014).

In the present study, we demonstrated that both peptides were effective in the treatment of cutaneous abscesses caused by *P. aeruginosa* (**Figure 4**). Using the moderately virulent PAO1 strain as well as the clinical isolate LESB58, we showed that peptide administration decreased skin lesion sizes and reduced bacterial loads inside the abscess – a phenotype resembling an untreated infection by the stringent response mutants. These observations led us to speculate that synthetic peptides can be used to target the *P. aeruginosa* stringent response *in vivo*. In this context, a partially restored *spoT* knockout (i.e., a *relA* expressing strain in the Δ*relA*/Δ*spoT* double mutant background) reduced susceptibility to peptide administration, which could be consistent with binding of the peptide to ppGpp, thereby reducing the effect of the peptide. However, it is important to point out that working with intact cells has its limitations and we only demonstrated *ex vivo* but not *in vivo* binding of the peptides to ppGpp. Moreover, since a *P. aeruginosa spoT* knockout is apparently not viable, the use of a partial complementation of the Δ*relA*/Δ*spoT* with the *relA* gene might have had unknown side effects that could also contribute to increased peptide resistance. The observation that treatment of the Δ*relA*/Δ*spoT* mutant with DJK-5 could still reduce abscess lesion sizes and bacterial loads recovered from the abscess tissue (**Figure 5**), indicated that the peptide DJK-5 might act not only on the stringent response, but might also target other bacterial cellular mechanisms such as interfering with cell wall synthesis, inhibition of DNA and protein synthesis, or interruption of protein folding and/or enzyme activity. Other possible impacts of peptide administration could be their known immunomodulatory activity as observed for example with 1018 in various animal models (Mansour et al., 2015).

Since an inability to maintain intracellular ppGpp homeostasis, as observed in strains overexpressing *relA*, leads to cell death, it was very interesting that we observed that synthetic peptides could suppress *spoT* promoter activity *in vivo* (**Figure 3**). Due to the decreased expression of the main enzyme that can hydrolyse ppGpp, we hypothesize that ppGpp homeostasis was disturbed, which might explain in part the attenuated virulence associated with peptide-treated infections. However, *spoT* is co-transcribed with the ω subunit *rpoZ* of the RNA polymerase, which could indicate that the observed effect is not *spoT*-specific. The ω subunit aids in the binding of the sigma factor to the polymerase that further influences gene expression (Gunnelius et al., 2014) and also might directly interact with ppGpp (Chatterji et al., 2007). Chatterji et al. (2007) also proposed that *rpoZ* has a direct role in the expression of *relA* (Chatterji et al., 2007) and consequently on ppGpp levels inside the cells, although the physiological role of *rpoZ* during the stringent response is not well-defined.

Our data invite speculation that cells treated with synthetic peptides that target the stringent response *in vivo*, share common traits with stringent response Δ*relA*/Δ*spoT* mutants during infection. Furthermore, the observation that synthetic peptides suppressed expression of the *rpoZ*-*spoT* operon *in vivo* enabled us to propose the that the synthetic peptides 1018 and DJK-5 not only bind and degrade ppGpp, but also down-regulate the expression of RNA polymerase ω subunit and *spoT*, which leads to the down-regulation of *relA*, consequently shutting down ppGpp production. This would be a response loop that would severely weaken the pathogen, and would be consistent with additional treatment options, e.g., conventional antibiotics that are synergistic with these peptides (Reffuveille et al., 2014; de la Fuente-Núñez et al., 2015). It will be important in the future to better understand the underlying mechanisms.

## Conclusion

We have demonstrated that the synthetic cationic peptides 1018 and DJK-5 exhibit excellent promise to treat Gram-negative high-bacterial-density *P. aeruginosa* infections in a cutaneous abscess mouse model. *In vivo*, both peptides suppressed *spoT* promoter activity and reduced tissue lesions and the numbers of bacteria recovered from the infected tissues. Both peptides increased animal welfare and appeared to target the stringent stress response *in vivo*, while DJK-5 seemed to also act on additional targets, other than ppGpp, during the infection. These additional targets for DJK-5 activity *in vivo* should be addressed in the future. Given the fact that increasing antibiotic resistance is a global threat to human health, synthetic peptides offer a considerable potential as an alternative and/or adjunctive therapy for the treatment of bacterial infections and merit further research. Future studies should aim to utilize stringent response inhibitors in conjunction with antimicrobial therapy to reduce prescribed antibiotics and this will provide a contribution to the fight against growing antibiotic resistance.

## Author Contributions

Conceptualization: RH and DP; formal analysis: DP; funding acquisition: RH and DP; investigation: DP, HW, and MB; methodology: DP, HW, and MB; project administration: RH and DP; resources: RH; supervision: RH; validation: RH and DP; writing – original draft: RH and DP; writing – reviewing and editing: RH and DP.

## Conflict of Interest Statement

The peptides described here have been filed for patent protection, assigned to RH’s employer the University of British Columbia, and licensed to ABT Innovations, Inc. in which the University of British Columbia and RH have shares. The other authors declare that the research was conducted in the absence of any commercial or financial relationships that could be construed as a potential conflict of interest.
