# Supplementary material for: Synthetic Peptides to Target Stringent Response-Controlled Virulence in a Pseudomonas aeruginosa Murine Cutaneous Infection Model
Source: Front Microbiol. 2017 Sep 27;8:1867. doi: 10.3389/fmicb.2017.01867 (PMC5623667; doi:10.3389/fmicb.2017.01867)
Supplement: Supplementary file 1 [file Data_Sheet_1.docx]

**Supporting information**

# Table S1: Plasmids used in this study

| **Plasmid** | **Relevant characteristics^a^** | **Reference or source** |
| --- | --- | --- |
| pUC18-mini-Tn7 | Gm^r^, transposon | ([Choi and Schweizer, 2006](#_ENREF_8)) |
| pTNS3 | Ap^r^, Helper plasmid encoding Tn7 site-specific genes | ([Choi et al., 2008](#_ENREF_7)) |
| pRK2013 | Km^r^, Helper plasmid for conjugation | ([Figurski and Helinski, 1979](#_ENREF_18)) |
| pTOPO-pCR-BluntII | Km^r^, Zero Blunt TOPO vector | Invitrogen |
| pUC18T-mini-Tn7-*lux*-Gm | Gm^r^, suicide vector for single gene chromosomal insertion via mini-Tn7 element; lux under *P1* integron promoter | ([Damron et al., 2013](#_ENREF_11)) |
| pUC-mini-Tn7-*16S*-Pro | Gm^r^, *P1* integron promoter replaced by *16S* promoter region (PAO1) on pUC18T-mini-Tn7-*lux*-Gm | This study |
| pUC-mini-Tn7-*relA*-Pro | Gm^r^, *P1* integron promoter replaced by *relA* promoter region (PAO1) on pUC18T-mini-Tn7-*lux*-Gm | This study |
| pUC-mini-Tn7-*spoT*-Pro | Gm^r^, *P1* integron promoter replaced by *spoT* promoter region (PAO1) on pUC18T-mini-Tn7-*lux*-Gm | This study |
| pUC18-mini-Tn7-*relA*(PAO) | Gm^r^, contains the 2.4-kb *relA* gene and its promoter region from PAO1 | This study |
| pUC18-mini-Tn7-*spoT*(PAO) | Gm^r^, contains the 2.5-kb *rpoZ*-*spoT* operon and its promoter region from PAO1 | This study |
| pUC18-mini-Tn7-*relA*(LESB) | Gm^r^, contains the 2.4-kb *relA* gene and its promoter region from LESB58 | This study |
| pUC18-mini-Tn7-*spoT*(LESB) | Gm^r^, contains the 2.5-kb *rpoZ*-*spoT* operon and its promoter region from LESB58 | This study |
| pEX18Gm | Gm^r^, gene replacement vector | ([Hoang et al., 1998](#_ENREF_30)) |
| pEX18Gm.relA(PAO)-ko | Gm^r^, contains a 1.0-kb fusion fragment of the upstream and downstream region surrounding *relA* from PAO1 | This study |
| pEX18Gm.spoT(PAO)-ko | Gm^r^, contains a 1.0-kb fusion fragment of the upstream and downstream region surrounding *spoT* from PAO1 | This study |
| pEX18Gm.relA(LESB)-ko | Gm^r^, contains a 1.0-kb fusion fragment of the upstream and downstream region surrounding *relA* from LESB58 | This study |
| pEX18Gm.spoT(LESB)-ko | Gm^r^, contains a 1.0-kb fusion fragment of the upstream and downstream region surrounding *spoT* from LESB58 | This study |

^a^ Antibiotic resistance: Gm^r^, gentamicin, Tc^r^, tetracycline, Km^r^, kanamycin.

**Table S2: Primers used in this study.**

| **Primer** | **Sequence (5' - 3')** |
| --- | --- |
| **Knockout primers** |  |
| relA-up-F1 | CCGGATCGGCGGGCTGGGCAATTTCTCCCTGCCGCTGGCG |
| relA-up-R1 | GCCCGTGGCCTGTTTCCGCCTCGCCCTTGCCTACCCTTTACCACGGTGCG |
| relA-down-F2 | CGCACCGTGGTAAAGGGTAGGCAAGGGCGAGGCGGAAACAGGCCACGGGC |
| relA-down-R2 | GGCTGCAGCGGCACGCTCCTCGGCCTTCAGTTCTTCCC |
| relA-up-R1LES | GCCCGTGGCCTGTTTCCACCTCGCCCTTGCCTACCCTTTACCACGGTGCG |
| relA-down-F2LES | CGCACCGTGGTAAAGGGTAGGCAAGGGCGAGGTGGAAACAGGCCACGGGC |
| spoT-up-F1 | CCGGATCCGCGATCTTCCGCGCCCGCCAGTTGC |
| spoT-up-R1 | GGGCGCCGGATGACACAGGAAAAAGCGGGGGGTTCACCCCCTGCCCGTAGGCGG |
| spoT-down-F2 | CCCGCCTACGGGCAGGGGGTGAACCCCCCGCTTTTTCCTGTGTCATCCGGCGCCC |
| spoT-down-R2 | GGCTGCAGGCATCGTGCTACTCCATGCAGAGCGACGGG |
| relA_out1 | CTGGTCCTGGTGCCGCCTTTGC |
| relA_out2 | CCTTGAGATGCCGGGCCAGGTTGACCACG |
| spoT_out3 | AGGACAGCGACGAGGTGAT |
| spoT_out4 | CTTCCATTCCAGGTTCGGG |
| **Complementation** |  |
| rpoZ-Pro_fwd | CCAGGGCCCGAGCTCGTCTGCTCGCCTAAATCGG |
| spoT_rev | CGAGGTACCGGAAAAAGCGGGTCAGCTACG |
| relA-Pro_fwd | CATACTAGTGATGCCTGCGTAATCCGA |
| relA_rev | TCAGGGCCCGCTAGGATGCCTGCGTAATC |
| Tn7L | ATTAGCTTACGACGCTACACCC |
| Tn7R | CACAGCATAACTGGACTGATTTC |
| glmS_up | CTGTGCGACTGCTGGAGC |
| glmS_down | GCACATCGGCGACGTGCTCTC |
| **Promoter fusions** |  |
| 16S-Pro_fwd | CATGGATCCGCATCACGCTGAAAGCTGA |
| 16S-Pro_rev | TTCCTGCAGCTTCAGTTCAATACTGCTTGGG |
| rpoZ-Pro_fwd | AGTGGATCCGTCTGCTCGCCTAAATCGG |
| rpoZ-Pro_rev | TTCCTGCAGGTGTTCCTCGTAACGAAAAGTG |
| relA-Pro_fwd | AGTGGATCCGATGCCTGCGTAATCCGA |
| relA-Pro_rev | TTCCTGCAGCTTGCCTACCCTTTACCACG |


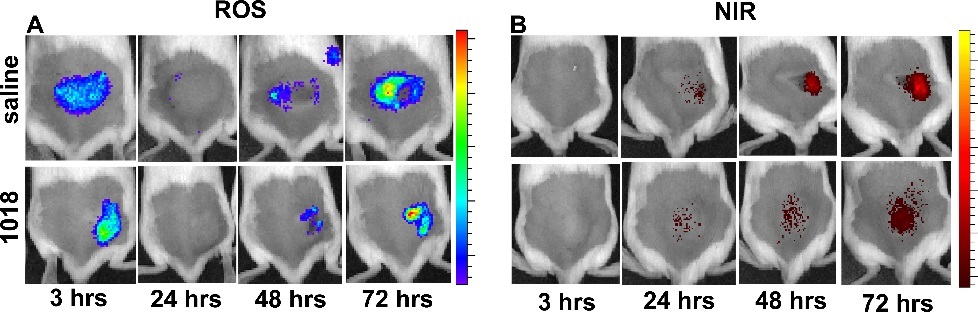


**Fig S1: *In vivo* tracking of reactive oxygen and nitrogen species and neutrophil activation.** The infection progress was monitored as indicated. Female CD-1 mice were injected with a high bacterial number (5×10^7^ LESB58) and subsequently (1 hour post infection) treated with either saline (control) or peptide 1018. (A) ROS and RNS production was tracked using the chemiluminescent probe L-012 (injected between the ears). Radiance color scale from 0.5 to 2.5 x 10^5^. (B) Mice were injected intravenously with a fluorescent neutrophil specific NIR probe. Radiant efficiency scale from 0.2 to 1.8 x 10^6^ (A,B) Mice were imaged using the in vivo image system (IVIS). The experiment was repeated at least twice times with a minimum of three mice / group.
